# Multifunctional Viologen-Derived Supramolecular Network with Photo/Vapochromic and Proton Conduction Properties

**DOI:** 10.3390/molecules26206209

**Published:** 2021-10-14

**Authors:** Chuanqi Zhang, Huaizhong Shi, Chenghui Zhang, Yan Yan, Zhiqiang Liang, Jiyang Li

**Affiliations:** 1 State Key Lab of Inorganic Synthesis and Preparative Chemistry, Jilin University, Changchun 130012, China; cq.zhang1@siat.ac.cn (C.Z.); shihz16@mails.jlu.edu.cn (H.S.); mse_zhangch@ujn.edu.cn (C.Z.); yanyan@jlu.edu.cn (Y.Y.); liangzq@jlu.edu.cn (Z.L.); 2 Shenzhen Institute of Advanced Electronic Materials, Shenzhen Institutes of Advanced Technology, Chinese Academy of Sciences, Shenzhen 518055, China; 3 Laboratoire Catalyse et Spectrochimie (LCS), Normandie University, ENSICAEN, CNRS, 6 Boulevard du Marechal Juin, 14050 Caen, France

**Keywords:** supramolecular network, viologen ligand, photo/vapochromism, proton conductivity

## Abstract

A supramolecular network [H_4_bdcbpy(NO_3_)_2_·H_2_O] (H_4_bdcbpy = 1,1′-Bis(3,5-dicarboxybenzyl)-4,4′-bipyridinium) (**1**) was prepared by a zwitterionic viologen carboxylate ligand in hydrothermal synthesis conditions. The as-synthesized (**1**) has been well characterized by means of single-crystal/powder X-ray diffraction, elemental analysis, thermogravimetric analysis and infrared and UV-vis spectroscopy. This compound possesses a three-dimensional supramolecular structure, formed by the hydrogen bond and π–π interaction between the organic ligands. This compound shows photochromic properties under UV light, as well as vapochromic behavior upon exposure to volatile amines and ammonia, in which the electron transfer from electron-rich parts to the electron-deficient viologen unit gives rise to colored radicals. Moreover, the intensive intermolecular H-bonding networks in **1** endows it with a proton conductivity of 1.06 × 10^−3^ S cm^−1^ in water at 90 °C.

## 1. Introduction

Crystalline chromic materials have attracted considerable attention for optical memory, visual monitoring, digital anti-counterfeit measures, and environmental detection [1,2,3]. Compared to traditional inorganic/organic chromic materials, the introduction of photoelectric organic motifs in crystal engineering is one of the most promising strategies to produce target photochromic materials due to their designable and controllable structures [4,5]. Viologens (4,4′-quartinized bipyridinium salts) and their derivatives are known to produce colored viologen radicals under light [6], electricity [7], heat or chemical stimuli [8] for photochromism, electrochromism and thermochromism; thus, they have been extensively used in chromic materials and devices [9,10]. It was noted that zwitterionic bipyridinium carboxylate ligands contain redox-active bipyridinium cationic groups as cores and carboxylate groups as building blocks. The presence of the electron donor (carboxylate) and acceptor (bipyridinium) on the same structural unit ensures the availability of chromic behaviors. Recently, bipyridinium carboxylate-based metal-organic frameworks (MOFs) have emerged and have been well-studied in relation to the merits of these intriguing photoelectrochemical properties [11,12,13]. For instance, Zhang et al. reported the incorporation of flexible bipyridinium carboxylate ligands into MOFs, which exhibited highly reversible rapid X-ray-induced photochromism in a broad temperature range [14]. In addition, chromic MOFs exhibiting a differentiable color response to relatively small primary/secondary/tertiary amines were prepared using rigid 4,4′-bipyridinium-1,1′-bis(phenylene-3-carboxylate) [15].

In contrast to the large number of chromic MOFs, supramolecular networks containing bipyridinium carboxylate unit are still rare [16,17], and the further research to understand the chromic mechanism and enhance the chromic properties of supramolecular networks is highly desirable [18]. On the other hand, supramolecular networks possessing hydrophilic groups (such as -COOH, -SO_3_H and -PO_3_H_2_) and well-defined hydrogen bond paths may be eligible proton conductors for hydrogen fuel-cell applications [19,20]. Nevertheless, the related reports on the crystalline supramolecular networks are inadequate [21].

**Scheme 1:**
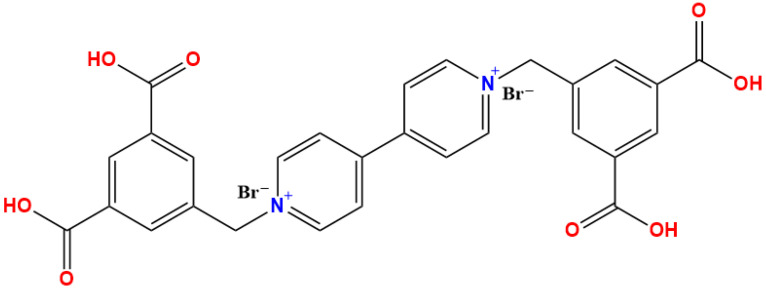
1,1′-bis(3,5-dicarboxybenzyl)-4,4′-bipyridinium dibromide (H_4_bdcbpy∙Br_2_).

**Scheme 2:**
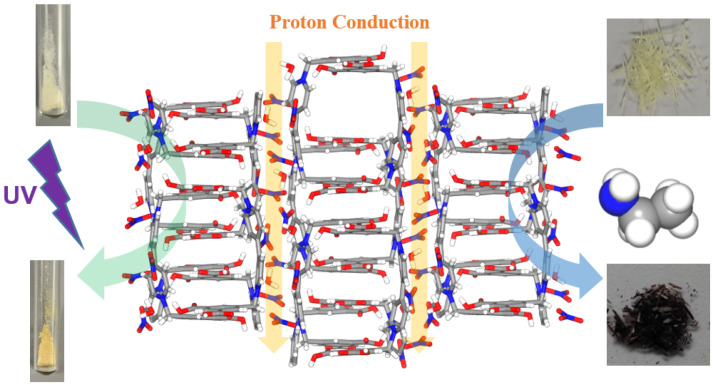
A viologen-based supramolecular framework with photo/vapochromic and proton conduction properties.

In this work, we report the structure of the supramolecular network assembled by flexible zwitterionic 1,1′-bis(3,5-dicarboxybenzyl)-4,4′-bipyridinium dibromide (H_4_bdcbpy∙Br_2_, displayed in Scheme 1), where the intermolecular hydrogen bond and π–π stacking interactions dominate the formation of the network. Compound **1** features photochromism upon UV light stimulation and vapochromism when fumed with electron-rich primary/secondary amines and ammonia. Meanwhile, compound **1** exhibits the proton conduction of 1.06 × 10^−3^ S cm^−1^ at 90 °C in water, as displayed in Scheme 2.

## 2. Results

### 2.1. Structural Description

**Figure 1:**
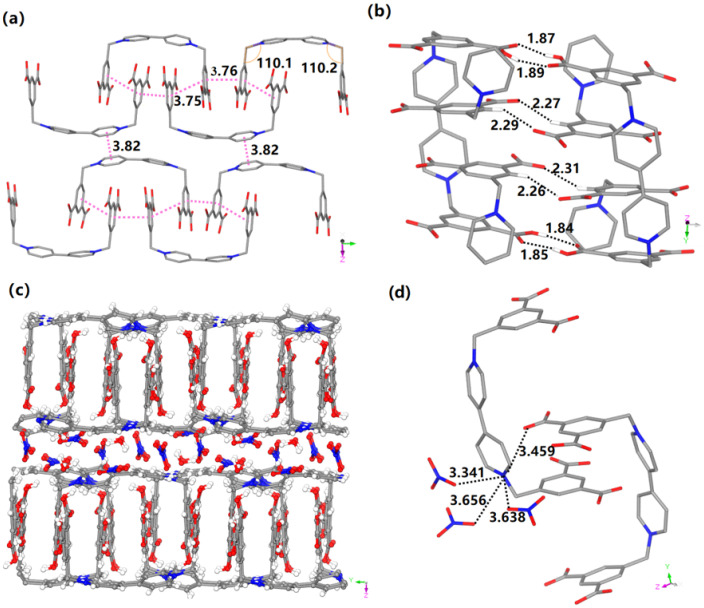
(**a**) The conformation of H_4_bdcbpy and the supramolecular layer formed by π–π type interactions (highlighted by the pink dotted lines). (**b**) The hydrogen bond interactions between the adjacent layers along *a* direction. (**c**) The free H_2_O and nitrate ions between the layers. (**d**) The O···N distance between adjacent carboxylate O atom, nitrate O atoms and bipyridinium N atom (Color modes: C—gray, N—blue, O—red and H—white. For clarity, some hydrogen atoms in (**a**,**b**,**d**) were omitted).

Compound **1** was prepared using the hydrothermal synthesis method, in which the combination of pressure and temperature facilitate the formation of crystal materials [22,23,24]. This compound crystallizes in the monoclinic crystal system and *P*2_1_/c space group (Table 1). In the asymmetric unit, it contains a H_4_bdcbpy molecule, two nitrate ions and one water molecule (Figure S1). Interestingly, the H_4_bdcbpy in the structure adapts the U-type cis-conformation with the dihedral angles of 110.13° and 110.20° between the bipyridinium unit and the isophthalate moiety (Figure 1a). As far as we know, the cis-conformation of this ligand has been rarely observed in other H_4_bdcbpy-based MOFs [25,26]. In the structure of **1**, the U-type H_4_bdcbpy molecules interdigitate each other up and down to assemble a supramolecular chain through the continuously alternating π–π type interactions with the centroid–centroid distance of 3.75 and 3.76 Å alternatively along the *b* axis (Figure 1a). These chains were further assembled to a layer through π–π stacking, with the distance of 3.82 Å between the adjacent bipyridinium units. Finally, a three-dimensional supramolecular network was formed by the intensive O–H···O hydrogen bonds between the lateral carboxylic groups along *a* direction (Figure 1b), and information on the lengths and the angles of the hydrogen bonds are listed in Table S2 in the Supporting Information (SI). It is noteworthy that there are free H_2_O and nitrate counter anions between the molecular layers (Figure 1c). The existence of these guest molecules not only generates a continuous hydrogen bond pathway with bipyridinium carboxylate ligands but also can exert an important impact on its inherent properties [27]. 

### 2.2. Proton Conduction Properties

**Figure 2:**
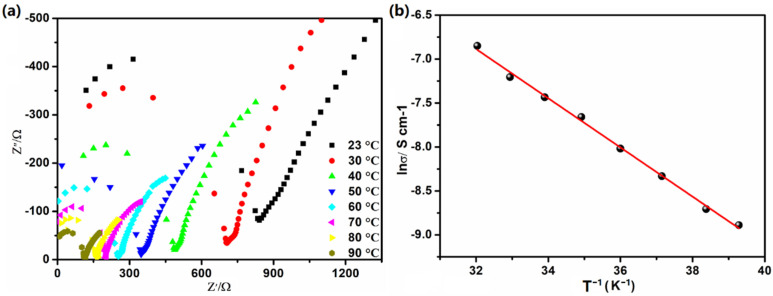
(**a**) The A C impedance plots and (**b**) an Arrhenius-type plot of compound **1** at different temperatures in water.

Encouraged by previous work on intensive intermolecular H-bonding networks, which may provide a proton transport pathway [20,28,29], we investigated the proton conduction properties of compound **1** by means of AC impedance spectroscopy in water. A compacted pellet sample of **1** was prepared and measured for impedance analyses; more details on the conductivity measurements are provided in Section 3.2. As revealed in the AC impedance plots shown in Figure 2a, the proton conductivity of compound **1** at 23 °C is 1.38 × 10^−4^ S cm^−1^. Furthermore, the proton conductivity of **1** presents a temperature-dependent trend. It increases to 1.06 × 10^−3^ S cm^−1^ when the temperature is raised to 90 °C. It is remarkable that the PXRD of compound **1** maintains its structure after the proton conduction test in water, which indicates the moderate hydrothermal stability of this compound (Figure S2). In addition, thermogravimetric analysis (TGA) shows that this compound can remain stable up to 200 °C. Crystal lattice H_2_O and nitrate counter anions are eliminated between 200 °C and 260 °C, and then the ligand starts to decompose upon further heating (Figure S3). As is well known, the activation energy (*E*a) for the proton transfer can be calculated using Arrhenius plots (ln(σT) vs. 1000 T^−1^) to figure out the proton conduction mechanism [30]. The calculated *E*a value of compound **1** is 0.28 eV, in the range of 0.1–0.4 eV, demonstrating that the proton conduction process follows the Grotthuss mechanism. The proton conductivity of **1** is not comparable to the ultrahigh proton conductivity of hydrogen-based organic framework (HOF) materials HOF-GS-10 (0.75 × 10^−2^ S cm^−1^) and HOF-GS-11 (1.8 × 10^−2^ S cm^−1^) at 30 °C and 95% relative humidity (RH), respectively [31]. However, it is several orders of magnitude higher than other reported HOFs, such as HOF-H_3_L (6.91 × 10^−5^ S cm^−1^) at 100 °C and 98% RH [32], hydrated HOF-6a (3.4 × 10^−6^ S cm^−1^ at 300 K and ∼97 % RH) [21] and the pair of zwitterionic supramolecular structures of 2-phenylbenzimidazole-5-sulfonicacid, with magnitudes of 1 × 10^−5^ S cm^−1^ for the mono-hydrated form and 5 × 10^−5^ S cm^−1^ for the di-hydrated form [33]. The decent proton conductivity of compound **1** may be explained by the continuous hydrogen bond pathway established by the ligands, guest water molecules and nitrate ions, as discussed in the structural section (Figure 1b) [34].

### 2.3. Photo/Vapochromic Properties

**Figure 3:**
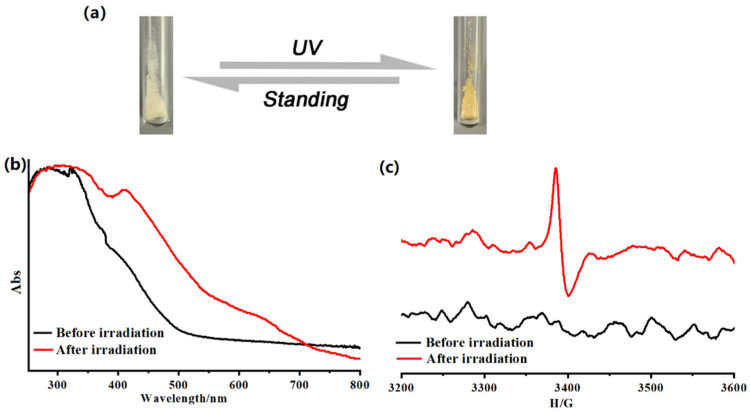
(**a**) Photographs, (**b**) Normalized UV−vis adsorption spectra and (**c**) EPR spectra of compound **1** before and after irradiation with a UV lamp.

In general, bipyridinium carboxylate ligands in the crystal structure may undergo a two-step redox process and exhibit three different states through the electron transfer from electron donors (carboxylate groups) towards electron acceptors (bipyridinium units), which is accompanied by a color change due to the formation of viologen radicals [35]. The photochromic behavior of compound **1** was studied for this reason, as shown in Figure 3a. The yellowish crystals become dark yellow after UV irradiation for 30 mins. The electron transfer process was verified by solid-state UV-vis adsorption spectra and electron paramagnetic resonance (EPR). As shown in Figure 3b, two new characteristic absorption bands around 420 and 630 nm appear after UV irradiation [17,36,37,38]. Moreover, an intensive signal with g = 2.00 occurs, indicating the generation of viologen radicals in **1** during the photochromic process (Figure 3c). It has been well recognized that the distance between electron donors and acceptors plays an essential role in determining the efficiency of photochromism [39]. As shown in Figure 1d, in compound **1**, the closest O···N distance between the adjacent oxygen atoms of nitrate, carboxylate O atoms of the isophthalate moiety and the nitrogen atoms of the bipyridinium unit are 3.341 and 3.459 Å, respectively, which are in the range of UV-light-induced photochromism [40,41].

**Figure 4:**
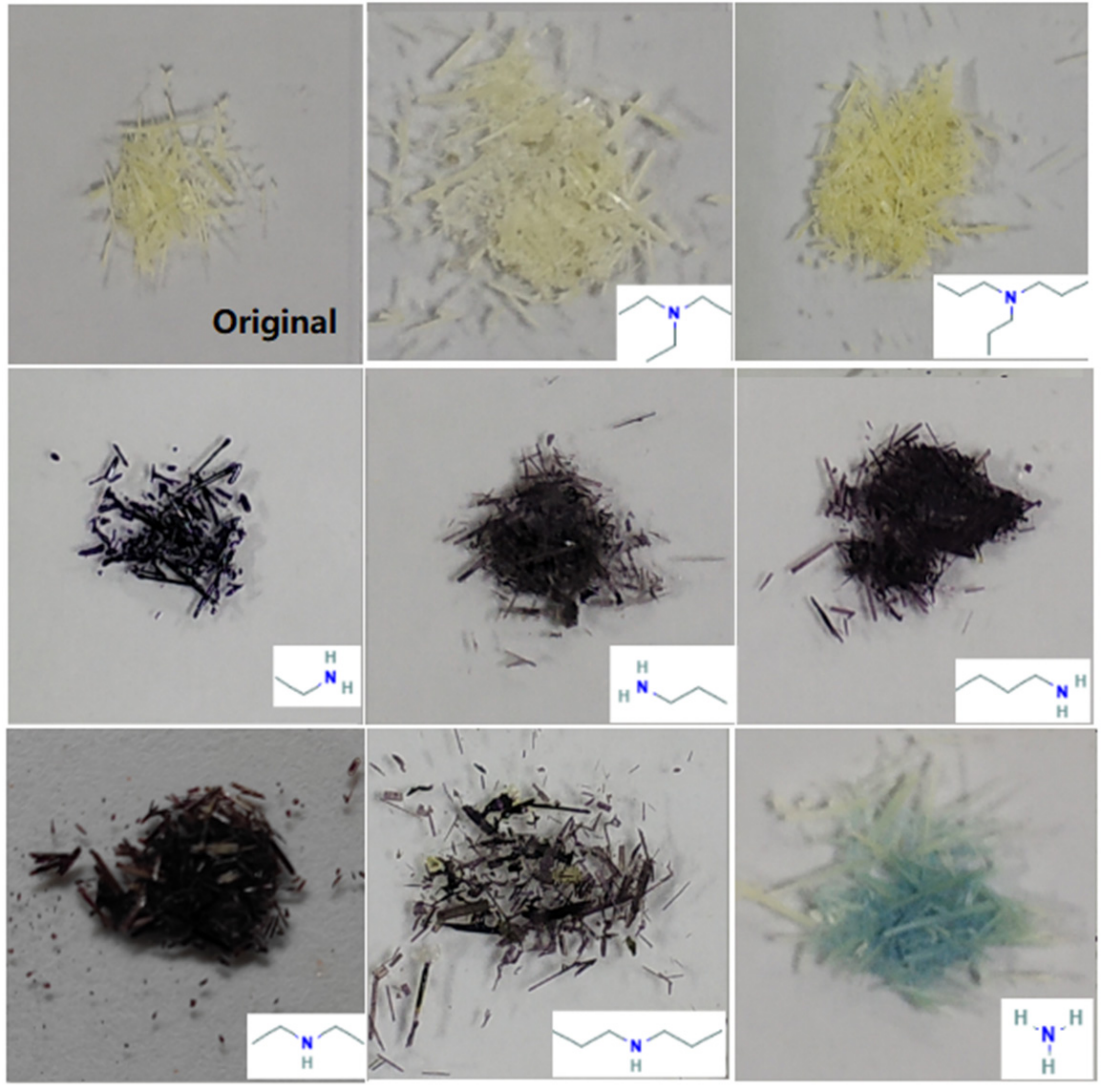
Photographs of compound **1** before and after exposure to different volatile amine vapors.

**Figure 5:**
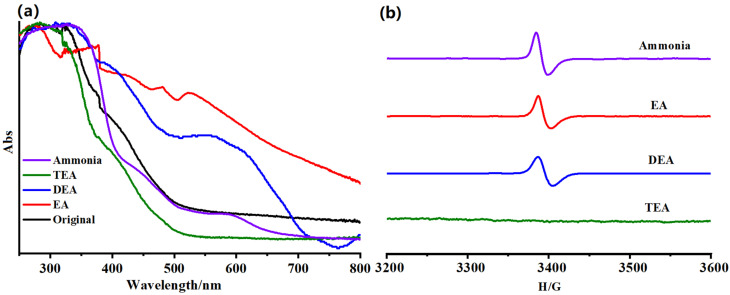
(**a**) Normalized UV−vis absorption spectra and (**b**) EPR spectra of compound **1** before and after exposure to different volatile amine/ammonia vapors, (EA = ethylamine, DEA = diethylamine and TEA = triethylamine).

The detection of volatile amines has attracted extensive attention because the widespread existence of these toxic, corrosive chemicals greatly threatens human health and the ecological system [42,43]. Chromic MOF materials are capable of detecting volatile organic compounds through a visual color response [44,45], we studied the sensing ability of compound **1** for amine vapors. Although the yellowish compound **1** has a negligible response to tertiary amines (e.g., triethylamine (TEA) and tri-n-propylamine (TPA)), it shows different color responses to primary/secondary amines and ammonia. As pictured in Figure 4, compound **1** turns deep purple immediately when exposed to the vapor of primary amine (e.g., ethylamine (EA), n-propylamine (PA) and n-butylamine (BA)) and secondary amine (e.g., diethylamine (DEA) and dipropylamine (DPA)). Moreover, fuming the compound **1** with ammonia leads to the color light blue. The solid-state UV-vis and EPR spectra were performed to verify the vapochromic mechanism. As shown in Figure 5a, the deep purple samples fumed with EA and DEA show intensive absorptions in the visible region. The light blue sample resulting from ammonia exhibits a week adsorption around 600 nm, and there is no obvious absorption change in the spectra of the TEA-treated sample. Moreover, the EPR spectra of those vapochromic samples show single signals with g = 1.99 that can be ascribed to viologen radicals (Figure 5b). Thus, it is reasonable to suppose that the vapochromic mechanism occurs due to the formation of viologen radicals caused by electron transfer from electron-rich amines or ammonia to the viologen unit in **1**. Although thermal electron transfer from the amines to the viologen moiety seems unfavorable in compound **1**, the acid−base interaction between the ammonia/amines (Lewis base) and the viologen moiety (Lewis acidic site) may facilitate intermolecular electron transfer [43,46,47]. It was noteworthy that the interactions between ammonia/amines and compound **1** should be on its surface due to the absence of porosity in its structure. In addition, the three alkyl groups on the tertiary amines impose steric hindrance for the amino group to interact with the viologen unit of compound **1**, which could be the reason for the negligible responses of TEA and TPA [48]. Impressively, those vapochromic samples are able to turn back to their pristine yellowish form after standing in air at room temperature for several minutes, suggesting that the vapochromic behaviors are reversible.

## 3. Materials and Methods

### 3.1. Materials and General Methods

1,1′-Bis(3,5-dicarboxybenzyl)-4,4′-bipyridinium dibromide (H_4_bdcbpy·Br_2,_) was obtained according to the procedure in the literature [26], all the other chemical reagents were commercially obtained and used without further purification. Powder X-ray diffraction (PXRD) patterns were performed on a Rigaku D-Max 2550 diffractometer (Rigaku Corporation, Tokyo, Japan), using Cu-Kα radiation (*α* = 1.5418 Å) in a 2θ range of 4–40°. The elemental analysis was performed on a Perkin-Elmer 2400 elemental analyzer (PerkinElmer, Waltham, MA, USA). The infrared spectrum was collected in the range of 400–4000 cm^−1^ on a Nicolet 6700 FT-IR spectrometer (Thermo Scientific, Waltham, MA, USA). The UV-vis absorption spectra were recorded on a Shimadzu UV-2450 spectrophotometer (Shimadzu Corporation, Kyoto, Japan). Electron paramagnetic resonance (EPR) spectra were collected using a JEOL JES-FA200 EPR spectrometer (JEOL Ltd., Tokyo, Japan). Thermo-gravimetric analysis (TGA) was carried out on a Perkin-Elmer TGA-7 thermogravimetric analyzer (PerkinElmer, Waltham, MA, USA) from room temperature to 800 °C in air atmosphere at a heating rate of 10 °C min^−1^.

### 3.2. Conductivity Measurements

Proton conductivity analyses were measured via impedance spectroscopy on a Solartron 1260 + 1287 impedance analyzer. The powder samples of compound **1** were pressed into a wafer on a tablet machine under 10 GPa. Both sides of the obtained tablet sample were coated with silver glue and stuck with silver wire for the proton conductivity analyses. Impedance data of these samples were collected in water with temperatures ranging from 23 °C–90 °C, performed in a water bath. The ranges of the applied frequency and alternating current voltage were from 10 Hz to 1 MHz and 300–500 mV, respectively. It is worth mentioning that the test was completed within 40 minutes because the silver glue on both surfaces of the tablet sample would be warped if immersed in water for a long time. The conductivity was calculated using the following equation: σ = l/(Rs × S), where l and S are the thickness (cm) and cross-sectional area (cm^2^) of the pellet, respectively, and Rs was extracted directly from the impedance plots, indicating the bulk resistance of the sample (Ω). 

### 3.3. Crystal Structure Determination

Single crystal X-ray diffraction measurements were collected on a Bruker AXS SMART APEX II (Bruker, Karlsruhe, Germany) diffractometer for compound **1** with graphite monochromated Mo-Kα (λ = 0.71073 Å) radiation at 293 K. Data processing was performed using the SAINT processing program. The structure was solved through direct methods and refined on *F*^2^ by means of the full-matrix least-squares method with the SHELX-97 program. All the non-hydrogen atoms were refined with anisotropic thermal parameters. A summary of the detailed crystallographic data and structure refinement parameters for **1** (CCDC No. 2106882) are given in Tables S1–S3 in the SI.

### 3.4. Synthesis of [H_4_bdcbpy(NO_3_)_2_·H_2_O] (**1**)

30 mg of H_4_bdcbpy·Br_2_ was dispersed into 10 mL distilled water, then 0.85 mL of NaOH (1 M) and 1.5 mL of HNO_3_ (0.9 M) were added to obtain a clear solution. The mixture was sealed in a 20 mL glass bottle, and then heated at 100 °C for 1 day under static conditions. The light-yellow crystal was produced, washed by distilled water and dried at room temperature. As shown in Figure S2 in the SI, the experimental PXRD pattern of the as-made compound **1** matched well with the simulated one from its crystal structure, which confirms the purity of the phase. Figure S4, in the SI, displays the IR spectrum of compound **1**; the adsorption around 1354, 1631 and 3530 cm^−1^ may be assigned to the presence of nitrate ion and guest water molecule, respectively. The sharp adsorption peak near 3050 cm^−1^ can be attributed to the stretching vibrations of the aromatic C-H bond. Importantly, the stretching vibration peaks at 1710 and 3400 cm^−1^ of the carboxylic acid group indicate the organic ligand is not deprotonated. Elemental Anal. Calcd. for **1**: C_28_H_24_N_4_O_15_ (M = 656.52); C: 51.23, N:8.53, H:3.68. Found: C: 49.84, N:8.08, H:3.60%.

## 4. Conclusions

In summary, an organic supramolecular framework for compound **1** was constructed by means of intensive π–π stacking interactions and hydrogen bonds between the flexible bipyridinium tetradentate carboxylate ligand and guest molecules. Benefiting from the intermolecular H-bonding networks, in water at 90 °C, compound **1** has a high proton conductivity of 1.06 × 10^−3^ S cm^−1^, and its proton-conducting mechanisms have also been discussed. Meanwhile, compound **1** exhibits photochromic properties, changing from yellowish to dark yellow upon UV irradiation, and distinct vapochromic sensing for ammonia, primary/secondary amines. The results of UV-vis and EPR spectrum analyses indicate that the photochromism and vapochromic responses to ammonia/amines result from the formation of viologen radicals through reversible electron transfer. Compound **1** represents a rare supramolecular structure, featuring moderate hydrothermal stability, high proton conductivity and photo/vapochromic properties. This research proves that the utilization of zwitterionic bipyridinium carboxylate ligands to establish supramolecular networks offers a promising strategy for the synthesis of new multifunctional materials in sensors and fuel cells. Moreover, more researches on the in-depth mechanism are needed to obtain a better understanding of the vapochromic behaviors of supramolecular materials in the future.

## Figures and Tables

**Table 1:** Crystal data and structure refinement for compound **1**.

Empirical Formula	C_28_ H_24_ N_4_ O_15_
Formula weight	656.51
Temperature	293(2) K
Wavelength	0.71073 Å
Crystal system, space group	Monoclinic, *P*2_1_/c
	*a* = 16.653(3) Å *α* = 90°
Unit cell dimensions	*b* = 13.379(2) Å *β* = 98.486(3)°
	*c* = 24.101(4) Å *γ* = 90°
Volume	5310.9(15) Å^3^
Z	8
Absorption coefficient	1.36 mm^−1^
*F* (000)	2720
Theta range for data collection	1.26 to 28.31°
Limiting indices	−22 ≤ *h* ≤ 11, −17 ≤ *k* ≤ 17, −31 ≤ *l* ≤ 32
Reflections collected/unique	37,987
*R*_int_	0.0943
Completeness to theta = 28.31	99.4%
Absorption correction	Semi-empirical from equivalents
Max. and min. transmission	0.9759 and 0.9720
Refinement method	Full-matrix least-squares on *F*^2^
Data/restraints/parameters	13,137/4/851
Goodness-of-fit on *F*^2^	1.004
Final R indices [*I* > 2σ(*I*)]	*R*_1_ = 0.0859, *wR*_2_ = 0.2112
*R* indices (all data)	*R*_1_ = 0.1813, *wR*_2_ = 0.2675
Largest diff. peak and hole	0.749 and −0.555 e Å^−3^

*R*_1_ = Σ||*F_o_*| − |*F_c_*||/Σ|*F_o_*|. *wR*_2_ = [Σ[*w* (*F*_o_^2^ − *F*_c_^2^)^2^]/Σ[*w* (*F*_o_^2^)^2^]]^1/2^.

## Data Availability

The data presented in this study are contained within the article.

## Supplementary Materials

The following Supporting Information is available online, Figure S1: The asymmetric unit of the structure for compound **1**; Figure S2: The PXRD patterns of simulated and as-synthesized compound **1**; Figure S3: The TG curve of compound **1** determined in the air atmosphere. Figure S4: The infrared spectrum of compound **1**. Selected bond lengths and angles and hydrogen bonds for compound **1** are provided in Tables S1 and S2.

## Sample Availability

Sample of the compound **1** is available from the authors.
