# Supplementary material for: Multifunctional Viologen-Derived Supramolecular Network with Photo/Vapochromic and Proton Conduction Properties
Source: Molecules. 2021 Oct 14;26(20):6209. doi: 10.3390/molecules26206209 (PMC8538028; doi:10.3390/molecules26206209)
Supplement: Supplementary file 1 [file molecules-26-06209-s001.zip › molecules-1395336-supplementary.pdf]

Multifunctional Viologen-Derived Supramolecular Network with photo/vapochromic and proton conduction properties

Chuanqi Zhang,[1,2,3+] Huaizhong Shi,[1+] Chenghui Zhang,[1] Yan Yan[1] Zhiqiang Liang[1] and Jiyang Li[1]*

1. State Key Lab of Inorganic Synthesis and Preparative Chemistry, Jilin University, Changchun 130012, P. R. China. E-mail: lijiyang@jlu.edu.cn.
2. Shenzhen Institute of Advanced Electronic Materials, Shenzhen Institutes of Advanced Technology, Chinese Academy of Sciences, Shenzhen 518055, China.
3. Laboratoire Catalyse et Spectrochimie (LCS), Normandie University, ENSICAEN, CNRS, 6 boulevard du Marechal Juin, 14050 Caen, France.
[+] These authors contributed equally to this work.

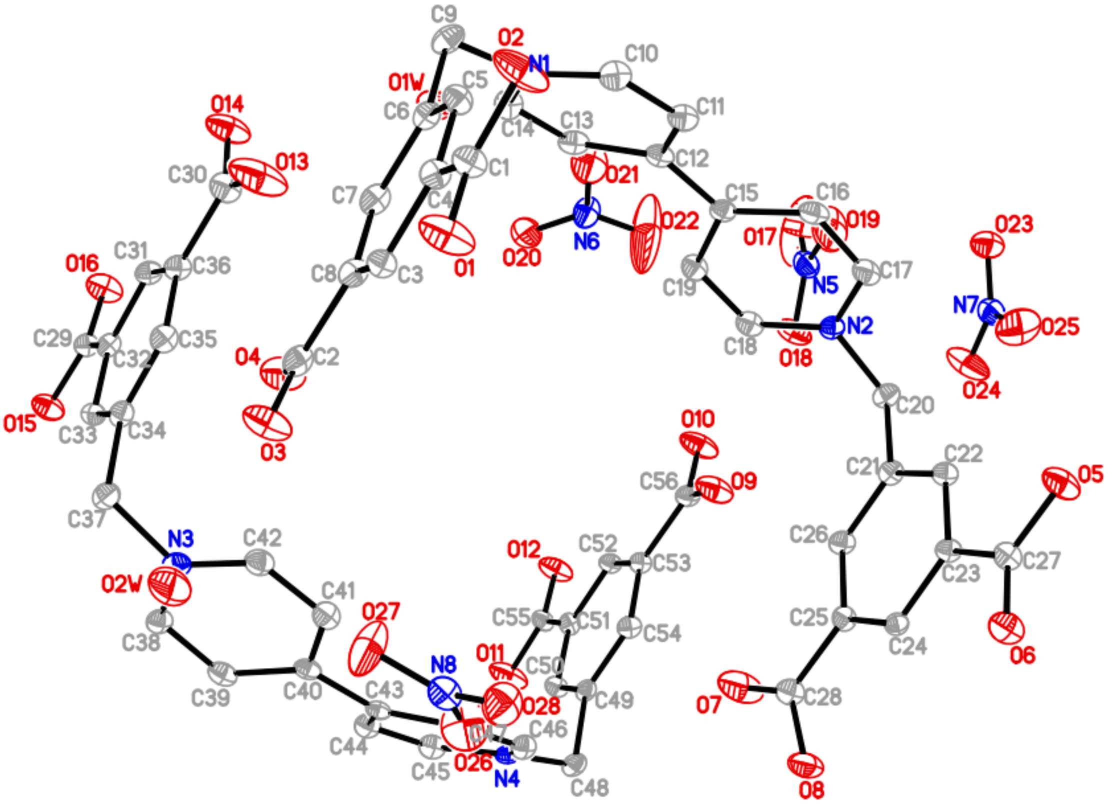

Figure S1. The asymmetric unit of the structure for compound **1**.

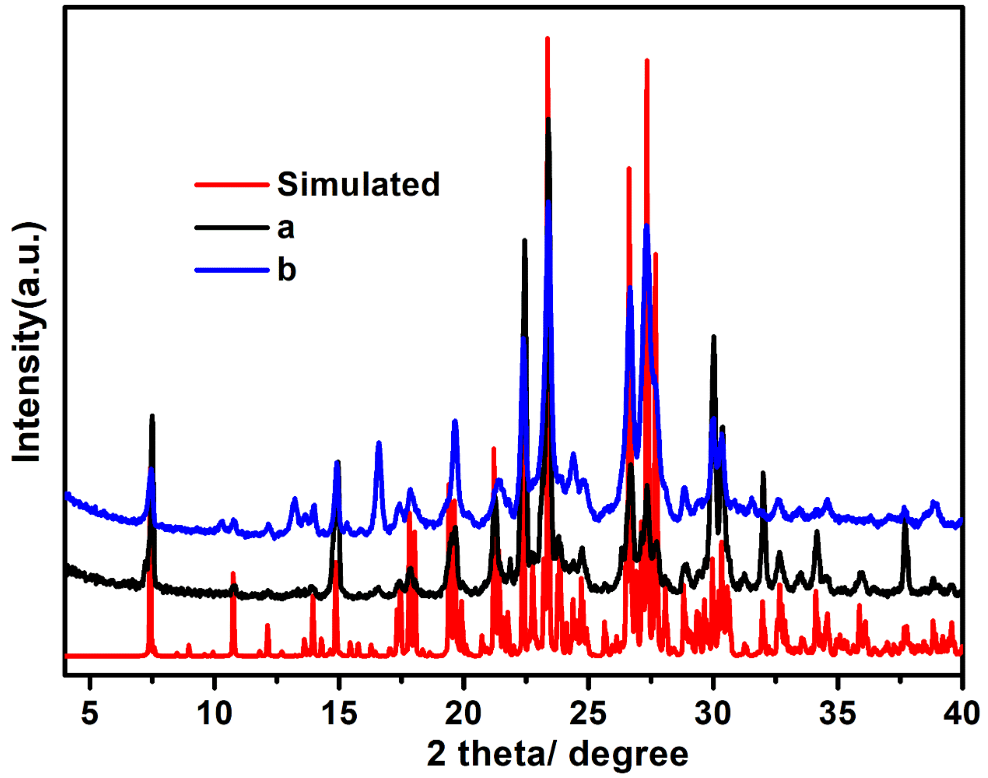

Figure S2. The PXRD patterns of simulated from the single-crystal structure of **1**, and the as-synthesized sample (a) and the sample after the proton conduction test at 90 °C in water.

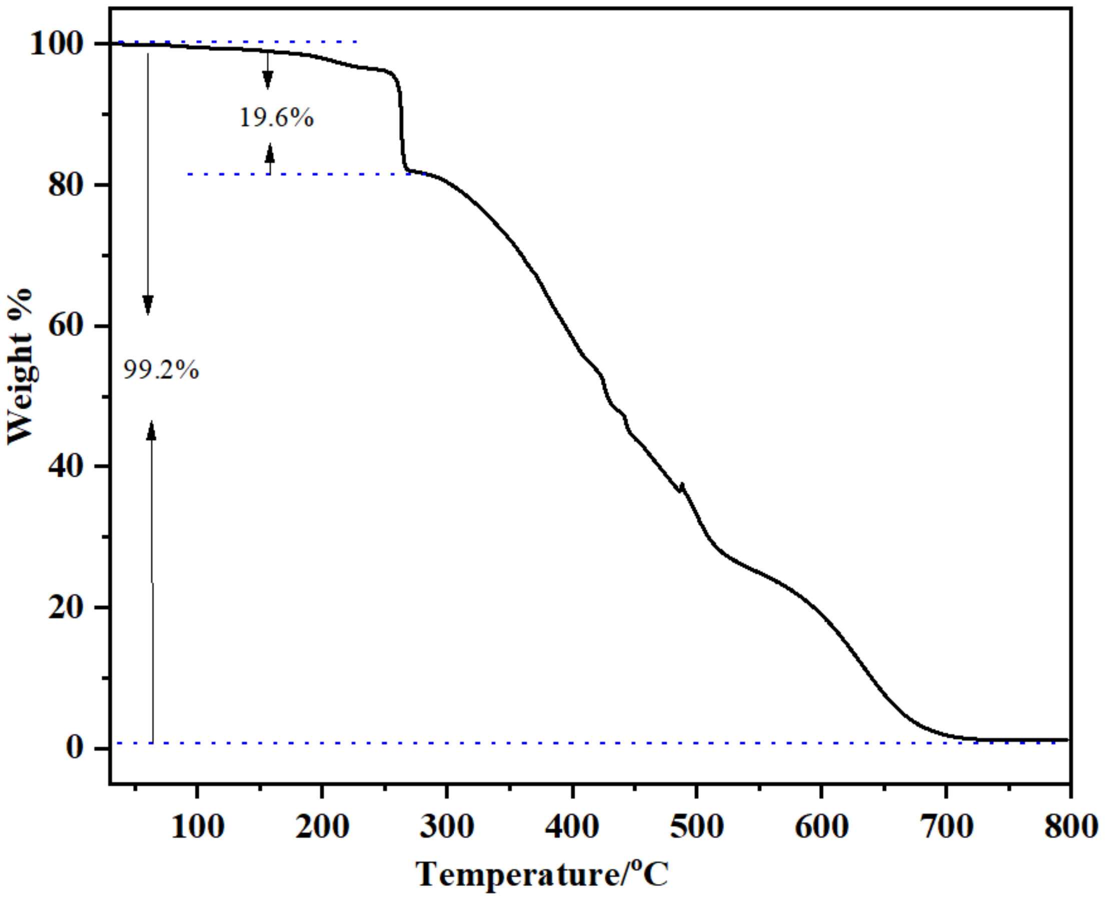

Figure S3. TG curve of the compound **1** performed in air atmosphere. The first weight loss of 19.6 wt% between 200-260°C corresponds to the removal of guest molecules (calcd. 21.5%), the ligand starts to decompose up to 260°C.

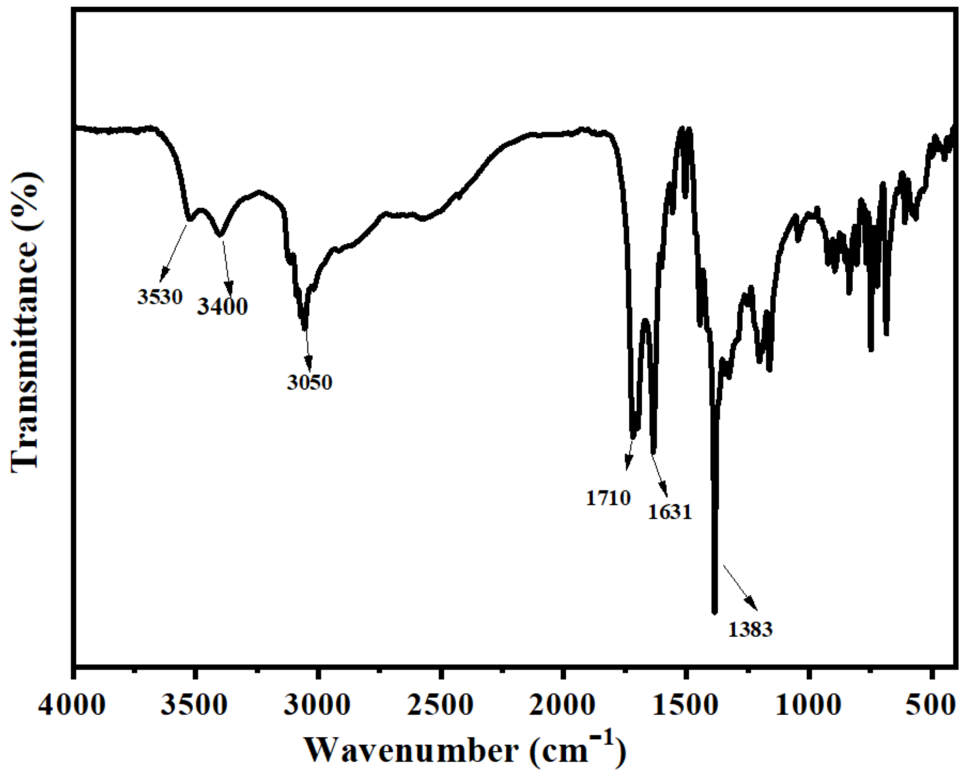

Figure S4. The Infrared spectrum of compound **1**.

Table S1 Selected bond lengths [Å] and angles [°] for compound **1**.

Bond lengths / Å

C(1)-O(1)	1.231(14)	C(22)-C(23)	1.398(15)
C(1)-O(2)	1.286(13)	C(22)-H(22)	0.9300
C(1)-C(4)	1.480(15)	C(23)-C(24)	1.388(14)
C(2)-O(4)	1.200(14)	C(23)-C(27)	1.489(15)
C(2)-O(3)	1.314(15)	C(24)-C(25)	1.381(15)
C(2)-C(8)	1.496(15)	C(24)-H(24)	0.9300
C(3)-C(8)	1.387(15)	C(25)-C(26)	1.400(15)
C(3)-C(4)	1.393(14)	C(25)-C(28)	1.493(15)
C(3)-H(3)	0.9300	C(26)-H(26)	0.9300
C(4)-C(5)	1.397(16)	C(27)-O(6)	1.236(13)
C(5)-C(6)	1.392(15)	C(27)-O(5)	1.291(12)
C(5)-H(5)	0.9300	C(28)-O(7)	1.195(14)
C(6)-C(7)	1.385(14)	C(29)-O(16)	1.229(13)
C(6)-C(9)	1.524(15)	C(29)-O(15)	1.294(12)
C(7)-C(8)	1.373(16)	C(29)-C(32)	1.494(15)
C(7)-H(7)	0.9300	C(30)-O(13)	1.192(15)
C(9)-N(1)	1.509(16)	C(30)-O(14)	1.296(15)
C(9)-H(9A)	0.9700	C(30)-C(36)	1.490(15)
C(9)-H(9B)	0.9700	C(31)-C(36)	1.388(15)
C(10)-N(1)	1.335(15)	C(31)-C(32)	1.394(14)
C(10)-C(11)	1.377(18)	C(31)-H(31)	0.9300
C(10)-H(10)	0.9300	C(32)-C(33)	1.382(15)
C(11)-C(12)	1.400(15)	C(33)-C(34)	1.399(15)
C(11)-H(11)	0.9300	C(33)-H(33)	0.9300
C(12)-C(13)	1.392(16)	C(34)-C(35)	1.394(15)
C(12)-C(15)	1.475(17)	C(34)-C(37)	1.523(15)
C(13)-C(14)	1.376(18)	C(35)-C(36)	1.385(16)
C(13)-H(13)	0.9300	C(35)-H(35)	0.9300
C(14)-N(1)	1.340(14)	C(37)-N(3)	1.504(16)
C(14)-H(14)	0.9300	C(37)-H(37A)	0.9700
C(15)-C(19)	1.394(15)	C(37)-H(37B)	0.9700
C(15)-C(16)	1.408(17)	C(38)-N(3)	1.341(14)
C(16)-C(17)	1.370(19)	C(38)-C(39)	1.365(18)
C(16)-H(16)	0.9300	C(38)-H(38)	0.9300
C(17)-N(2)	1.344(14)	C(39)-C(40)	1.395(16)
C(17)-H(17)	0.9300	C(39)-H(39)	0.9300

C(18)-N(2)	1.346(15)	C(40)-C(41)	1.394(15)
C(18)-C(19)	1.381(18)	C(40)-C(43)	1.489(17)
C(18)-H(18)	0.9300	C(41)-C(42)	1.375(18)
C(19)-H(19)	0.9300	C(41)-H(41)	0.9300
C(20)-N(2)	1.500(15)	C(42)-N(3)	1.346(15)
C(20)-C(21)	1.524(15)	C(42)-H(42)	0.9300
C(20)-H(20A)	0.9700	C(43)-C(47)	1.393(16)
C(20)-H(20B)	0.9700	C(43)-C(44)	1.401(15)
C(21)-C(26)	1.378(14)	C(44)-C(45)	1.367(18)
C(21)-C(22)	1.389(15)	C(44)-H(44)	0.9300
C(45)-N(4)	1.350(15)	N(5)-O(17)	1.171(17)
C(45)-H(45)	0.9300	N(5)-O(18)	1.191(15)
C(46)-N(4)	1.351(14)	N(5)-O(19)	1.226(15)
C(46)-C(47)	1.369(17)	N(6)-O(22)	1.180(19)
C(46)-H(46)	0.9300	N(6)-O(20)	1.213(14)
C(47)-H(47)	0.9300	N(6)-O(21)	1.244(15)
C(48)-N(4)	1.491(15)	N(7)-O(25)	1.227(15)
C(48)-C(49)	1.515(15)	N(7)-O(23)	1.231(14)
C(48)-H(48A)	0.9700	N(7)-O(24)	1.255(15)
C(48)-H(48B)	0.9700	N(8)-O(27)	1.230(16)
C(49)-C(54)	1.396(14)	N(8)-O(28)	1.233(14)
C(49)-C(50)	1.397(15)	N(8)-O(26)	1.238(15)
C(50)-C(51)	1.398(15)	O(1)-H(1)	0.8200
C(50)-H(50)	0.9300	O(3)-H(3A)	0.8201
C(51)-C(52)	1.394(14)	O(5)-H(5O)	0.8200
C(51)-C(55)	1.477(15)	O(8)-H(8O)	0.8200
C(52)-C(53)	1.384(15)	O(10)-H(10A)	0.8199
C(52)-H(52)	0.9300	O(11)-H(11O)	0.8200
C(53)-C(54)	1.394(15)	O(14)-H(14A)	0.8200
C(53)-C(56)	1.496(14)	O(16)-H(16O)	0.8200
C(54)-H(54)	0.9300	O(1W)-H(1W1)	0.9698
C(55)-O(12)	1.228(14)	O(1W)-H(2W2)	0.9813
C(55)-O(11)	1.288(13)	O(2W)-H(1W2)	0.9919
C(56)-O(9)	1.189(13)	O(2W)-H(2W1)	0.9749
C(56)-O(10)	1.317(14)		

Bond angles / °

O(1)-C(1)-O(2)	122.5(11)	C(3)-C(8)-C(2)	120.5(10)
O(1)-C(1)-C(4)	120.3(9)	N(1)-C(9)-C(6)	110.1(9)
O(2)-C(1)-C(4)	117.1(10)	N(1)-C(9)-H(9A)	109.6
O(4)-C(2)-O(3)	123.2(10)	C(6)-C(9)-H(9A)	109.6
O(4)-C(2)-C(8)	123.6(11)	N(1)-C(9)-H(9B)	109.6
O(3)-C(2)-C(8)	113.2(9)	C(6)-C(9)-H(9B)	109.6
C(8)-C(3)-C(4)	119.8(10)	H(9A)-C(9)-H(9B)	108.2
C(8)-C(3)-H(3)	120.1	N(1)-C(10)-C(11)	120.7(10)
C(4)-C(3)-H(3)	120.1	N(1)-C(10)-H(10)	119.7
C(3)-C(4)-C(5)	119.8(10)	C(11)-C(10)-H(10)	119.7
C(3)-C(4)-C(1)	118.3(10)	C(10)-C(11)-C(12)	120.0(11)
C(5)-C(4)-C(1)	121.9(9)	C(10)-C(11)-H(11)	120.0
C(6)-C(5)-C(4)	120.1(9)	C(11)-C(10)-H(10)	119.7
C(6)-C(5)-H(5)	119.9	C(10)-C(11)-C(12)	120.0(11)
C(4)-C(5)-H(5)	119.9	C(10)-C(11)-H(11)	120.0
C(7)-C(6)-C(5)	118.9(10)	C(6)-C(7)-H(7)	119.2
C(7)-C(6)-C(9)	121.4(10)	C(7)-C(8)-C(3)	119.8(10)
C(5)-C(6)-C(9)	119.7(9)	C(7)-C(8)-C(2)	119.7(10)
C(8)-C(7)-C(6)	121.5(10)	C(3)-C(8)-C(2)	120.5(10)
C(8)-C(7)-H(7)	119.2	N(1)-C(9)-C(6)	110.1(9)
C(6)-C(7)-H(7)	119.2	C(12)-C(11)-H(11)	120.0
C(7)-C(8)-C(3)	119.8(10)	C(13)-C(12)-C(11)	116.9(11)
C(7)-C(8)-C(2)	119.7(10)	C(13)-C(12)-C(15)	122.7(10)
C(3)-C(8)-C(2)	120.5(10)	C(11)-C(12)-C(15)	120.3(10)
N(1)-C(9)-C(6)	110.1(9)	C(14)-C(13)-C(12)	120.6(11)
N(1)-C(9)-H(9A)	109.6	C(14)-C(13)-H(13)	119.7
C(6)-C(9)-H(9A)	109.6	C(12)-C(13)-H(13)	119.7
C(6)-C(7)-H(7)	119.2	N(1)-C(14)-C(13)	120.2(11)
C(7)-C(8)-C(3)	119.8(10)	N(1)-C(14)-H(14)	119.9
C(7)-C(8)-C(2)	119.7(10)	C(13)-C(14)-H(14)	119.9
C(19)-C(15)-C(12)	121.4(10)	N(2)-C(17)-H(17)	119.7
C(16)-C(15)-C(12)	121.4(10)	C(16)-C(17)-H(17)	119.7
C(17)-C(16)-C(15)	120.9(10)	N(2)-C(18)-C(19)	121.1(10)
C(17)-C(16)-H(16)	119.6	N(2)-C(18)-H(18)	119.5
C(15)-C(16)-H(16)	119.6	C(19)-C(18)-H(18)	119.5
C(18)-C(19)-C(15)	120.2(11)	C(21)-C(20)-H(20A)	109.6
C(18)-C(19)-H(19)	119.9	N(2)-C(20)-H(20B)	109.6
C(15)-C(19)-H(19)	119.9	C(21)-C(20)-H(20B)	109.6
C(26)-C(21)-C(20)	119.7(10)	C(21)-C(22)-C(23)	120.1(9)
O(6)#1-Cd(2)-O(1)#5	92.98(14)	C(19)-C(18)-C(21)	121.2(4)
N(4)-C(48)-H(48B)	109.4	O(10)-C(56)-C(53)	112.9(9)
C(49)-C(48)-H(48B)	109.4	C(10)-N(1)-C(14)	121.0(11)
H(48A)-C(48)-H(48B)	108.0	C(10)-N(1)-C(9)	118.3(9)

C(54)-C(49)-C(50)	119.3(10)	C(14)-N(1)-C(9)	120.6(10)
C(54)-C(49)-C(48)	120.2(10)	C(17)-N(2)-C(18)	120.4(11)
C(50)-C(49)-C(48)	120.5(9)	C(17)-N(2)-C(20)	119.1(10)
C(49)-C(50)-C(51)	120.3(9)	C(18)-N(2)-C(20)	120.2(9)
C(49)-C(50)-H(50)	119.9	C(38)-N(3)-C(42)	120.5(11)
C(51)-C(50)-H(50)	119.9	C(38)-N(3)-C(37)	119.6(10)
C(52)-C(51)-C(50)	119.8(10)	C(42)-N(3)-C(37)	119.8(9)
C(52)-C(51)-C(55)	118.2(10)	C(45)-N(4)-C(46)	120.3(10)
C(50)-C(51)-C(55)	122.1(9)	C(45)-N(4)-C(48)	118.8(9)
C(53)-C(52)-C(51)	120.2(10)	C(46)-N(4)-C(48)	120.8(9)
C(53)-C(52)-H(52)	119.9	O(17)-N(5)-O(18)	124.6(14)
C(51)-C(52)-H(52)	119.9	O(17)-N(5)-O(19)	119.4(13)
C(52)-C(53)-C(54)	120.1(9)	O(18)-N(5)-O(19)	116.0(13)
C(52)-C(53)-C(56)	121.7(10)	O(22)-N(6)-O(20)	118.8(13)
C(54)-C(53)-C(56)	118.2(9)	O(22)-N(6)-O(21)	118.2(13)
O(25)-N(7)-O(23)	120.9(11)	C(2)-O(3)-H(3A)	109.3
O(25)-N(7)-O(24)	120.7(11)	C(27)-O(5)-H(5O)	109.5
O(23)-N(7)-O(24)	118.3(11)	C(28)-O(8)-H(8O)	108.3
O(27)-N(8)-O(28)	119.2(12)	C(56)-O(10)-H(10A)	109.3
O(27)-N(8)-O(26)	120.4(12)	C(55)-O(11)-H(11O)	109.5
O(28)-N(8)-O(26)	120.4(12)	C(30)-O(14)-H(14A)	109.2
C(1)-O(1)-H(1)	109.5	C(29)-O(16)-H(16O)	109.5
O(25)-N(7)-O(23)	120.9(11)	H(1W1)-O(1W)-H(2W2)	95.8
O(25)-N(7)-O(24)	120.7(11)	H(1W2)-O(2W)-H(2W1)	82.9

Table S2. Hydrogen bonds for compound **1** [Å and °].

D-H...A	d(D-H)	d(H...A)	d(D...A)	<(DHA)
O(11)-H(11O)...O(6)#1	0.82	1.84	2.653(12)	172.3
O(14)-H(14A)...O(18)#2	0.82	2.47	3.237(18)	156.2
O(16)-H(16O)...O(2)#1	0.82	1.89	2.637(12)	150.9
O(16)-H(16O)...O(1)#1	0.82	2.64	3.295(11)	137.3
O(1)-H(1)...O(15)#3	0.82	1.87	2.649(12)	158.4
O(1)-H(1)...O(16)#3	0.82	2.59	3.295(11)	144.4
O(5)-H(5O)...O(12)#3	0.82	1.85	2.668(11)	172.6
O(8)-H(8O)...O(2W)#4	0.82	2.53	3.258(15)	149.3
O(1W)-H(1W1)...O(26)#5	0.97	2.06	2.990(14)	159.2
O(1W)-H(1W1)...O(24)#2	0.97	2.42	2.913(17)	110.7
O(2W)-H(1W2)...O(19)#6	0.99	2.24	2.856(18)	118.6
O(2W)-H(2W1)...O(21)#6	0.97	2.29	2.985(15)	127.2
O(2W)-H(1W2)...O(22)#6	0.99	2.51	3.39(2)	148.0
O(2W)-H(2W1)...O(27)	0.97	2.12	2.848(17)	129.8
O(2W)-H(2W1)...O(21)#6	0.97	2.29	2.985(15)	127.2
O(1W)-H(2W2)...O(21)	0.98	1.85	2.817(17)	168.2
O(1W)-H(2W2)...N(6)	0.98	2.66	3.510(16)	145.1

Symmetry transformations used to generate equivalent atoms:

#1 x+1,y,z #2 x,y-1,z #3 x-1,y,z #4 x,y+1,z

#5 x,-y+1/2,z-1/2 #6 x,-y+1/2,z+1/2
